# Fitness cost of reassortment in human influenza

**DOI:** 10.1371/journal.ppat.1006685

**Published:** 2017-11-07

**Authors:** Mara Villa, Michael Lässig

**Affiliations:** Institute for Theoretical Physics, University of Cologne, Cologne, Germany; University of Texas at Austin, UNITED STATES

## Abstract

Reassortment, which is the exchange of genome sequence between viruses co-infecting a host cell, plays an important role in the evolution of segmented viruses. In the human influenza virus, reassortment happens most frequently between co-existing variants within the same lineage. This process breaks genetic linkage and fitness correlations between viral genome segments, but the resulting net effect on viral fitness has remained unclear. In this paper, we determine rate and average selective effect of reassortment processes in the human influenza lineage A/H3N2. For the surface proteins hemagglutinin and neuraminidase, reassortant variants with a mean distance of at least 3 nucleotides to their parent strains get established at a rate of about 10^−2^ in units of the neutral point mutation rate. Our inference is based on a new method to map reassortment events from joint genealogies of multiple genome segments, which is tested by extensive simulations. We show that intra-lineage reassortment processes are, on average, under substantial negative selection that increases in strength with increasing sequence distance between the parent strains. The deleterious effects of reassortment manifest themselves in two ways: there are fewer reassortment events than expected from a null model of neutral reassortment, and reassortant strains have fewer descendants than their non-reassortant counterparts. Our results suggest that influenza evolves under ubiquitous epistasis across proteins, which produces fitness barriers against reassortment even between co-circulating strains within one lineage.

## Author summary

The genome of the human influenza virus consists of 8 disjoint RNA polymer segments. These segments can undergo reassortment: when two viruses co-infect a host cell, they can produce viral offspring with a new combination of segments. In this paper, we show that reassortment within a given influenza lineage induces a fitness cost that increases in strength with increasing genetic distance of the parent viruses. Our finding suggests that evolution continuously produces viral proteins whose fitness depends on each other; reassortment reduces fitness by breaking up successful combinations of proteins. Thus, selection across proteins constrains viral evolution within a given lineage, and it may be an important factor in defining a viral species.

## Introduction

Influenza virus is a negative-sense single strand RNA virus. Humans can be infected by three phylogenetically and antigenically distinct influenza lineages—A, B and C—that co-circulate globally. Among these lineages, influenza A shows the fastest rate of evolution [1–4]. The genome of the virus is segmented into 8 RNA filaments that encode 11 different proteins. Within each segment, genomic evolution is a purely asexual process carried by point mutations [5], which are subject to genetic drift and natural selection. In particular, positive selective pressure by host immunity plays an important role in the evolution of the surface glycoproteins haemagglutinin (HA), which governs viral binding and entry into host cells, and neuraminidase (NA), which drives the release and escape of new virions from the cell [6, 7]. The gradual accumulation of adaptive mutations in these two proteins maintains the ability of the virus to continually evade host immunity [4, 8]; this phenotypic process has been called *antigenic drift* [9, 10].

In parallel to point mutations within single proteins, the genome of the influenza virus changes by so-called reassortment processes. If the same host cell is co-infected by two or more viruses carrying distinct genomes, mixing of genomic segments within that cell may produce a hybrid genotype carrying segments from different parental strains. The evolutionary implications of these dynamics are quite complex. On the one hand, in rare cases, reassortment can lead to *antigenic shifts*, which are new combinations of haemagglutinin and neuraminidase that strongly enhance fitness [11] by escape from host immunity [12, 13]. The acquisition of new HA and NA variants by human influenza A through reassortment with avian strains, for example, has been shown to cause global pandemics in 1957 and 1968, known as the “asian” and the “Hong Kong” flu, respectively [14, 15]. Many reassortments, however, have negligible antigenic effects but may have other fitness effects. Specifically, fitness interactions between segments across lineages are observed as biases in observed pairings [16–22]. By partly randomizing such pairings, reassortment generates a fitness cost and a resulting increase of subsequent compensatory mutations [23]. Broad negative selection has been postulated for reassortment between well distinct influenza B lineages [24], but the overall selective effects of intra-subtype ressortment have not been systematically analyzed so far.

In this paper, we infer a comprehensive map of intra-lineage reassortment between the surface proteins HA and NA of influenza A/H3N2, and we provide evidence that most of these events are under negative selection increasing with distance between parental strains. An important methodological basis for our analysis is a faithful inference of intra-subtype reassortment events from sequence data. Although these dynamics have long been recognized as a potentially important mechanism for evolution [25, 26], the detection of events within the same subtype is notoriously difficult due to their weak phylogenetic signal. There is a number of current methods to infer reassortment events from a data set of viral sequences. These methods can be roughly divided into two groups: distance-based methods [27, 28] and methods based on the phylogeny [13, 26, 29–35]. As recently pointed out [36], these approaches coherently report some fraction of the reassortment events but show a substantial degree of discrepancy between their results, which can be traced to method-specific differences in sensitivity. Distance-based methods rely on the assumption that, for reassortant strains, high similarity between the sequences of one segment goes along with large differences in the other segment. These methods do not pass through the step of inferring the phylogeny of the virus. They are fast, can be efficiently applied to large alignments, and are insensitive to errors in tree reconstruction. Without a viral phylogeny, however, it can be hard to determine if two or more inferred events are independent. Hence, distance-based methods can generate multiple representations of the same original event between unobserved ancestral strains. Resolving false positives constitutes a major issue for this kind of algorithms. Phylogenetic methods, on the other hand, are based on the observation that reassortant strains are located in different clades of the coalescent trees built for different segments. These approaches are usually successful in detecting reassortment across different lineages of the virus, i.e., between strains with substantial genetic differences. The incompatibility between tree topologies, however, can also be a result of phylogenetic errors, so that inconsistencies in the evolutionary histories of different segments are a necessary but not sufficient condition for reassortment. Successful attempts to overcome this issue [33] have produced algorithms which are applicable only to small datasets. Since the scaling of the number of inferred events with sample sizes has not been investigated, it is not clear if the rate of reassortment is independent on the size of the trees. Hence, even if the gain in information coming from the phylogenetic trees constitutes an advantage over the distance-based algorithms, the limited resolution constrains these methods in the detection of intra-lineage events [8]. In order to fill this methodological gap, we propose a new genealogical inference method for reassortment in fast-evolving populations with segmented genomes, such as influenza virus. We analyze the mutations arising in joint genealogies built from pairs of segments and set a simple criterion to identify reassortment events. An important part of our method is the exclusion of false positive events generated by ambiguities in tree reconstruction, which can be estimated from a statistical null model of non-reassorting sequences. In order to reduce the list of putative reassortments to a minimal set of independent events, we include internal inferred nodes as possible candidates for reassortment and cluster events with similar patterns of mutations. The overall number of reassortment events reported by our method turns out to be in broad agreement with the results of previous studies [37–39]. Furthermore, we have extensively tested our method on simulated data for evolving influenza-like strain populations under mutations, genetic drift, selection, and reassortment. We find that a large fraction of the simulated reassortment events are recovered by our algorithm, and these events outweigh the rate of false positives.

In the second part of the paper, we turn to our main biological objective of mapping selection on reassortment within an influenza lineage. We apply two independent and complementary selection inference methods to a set of intra-subtype reassortments in the A/H3N2 lineage inferred by our genealogy-based algorithm. First, we compare the distributions of the RNA distances between actual reassortant strains with a suitable background distribution of co-circulating strains, which quantifies the neutral opportunities for reassortment. Second, we compare the total population sizes between the reassortant clade and the non-reassortant clade defined by individual reassortment events; these sizes are estimated from the number of strains in the sequence sample. We find a consistent signal of broad negative selection on intra-lineage reassortment by both methods. We interpret this signal in terms of ubiquitous cross-protein epistasis and discuss evolutionary consequences.

## Results

### Reassortment events and their detection

**Fig 1:**
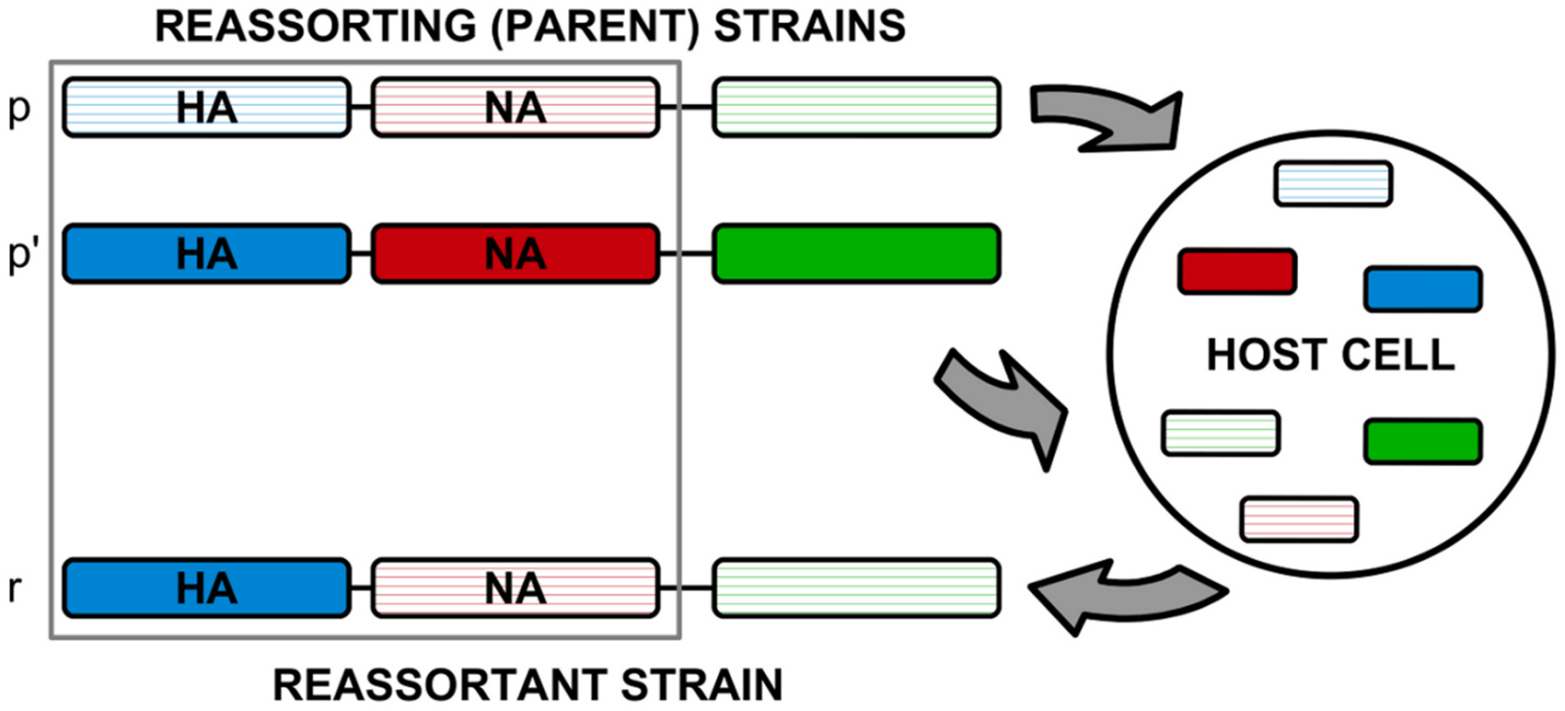
Schematic of a reassortment process. Two parent strains, *p* and *p*′, co-infect a host cell and produce a reassortant strain *r*. Here we focus on reassortment of the two surface proteins HA (blue segments) and NA (red segments); the reassortant strain *r* inherits one of these segments from each parent.

**Fig 2:**
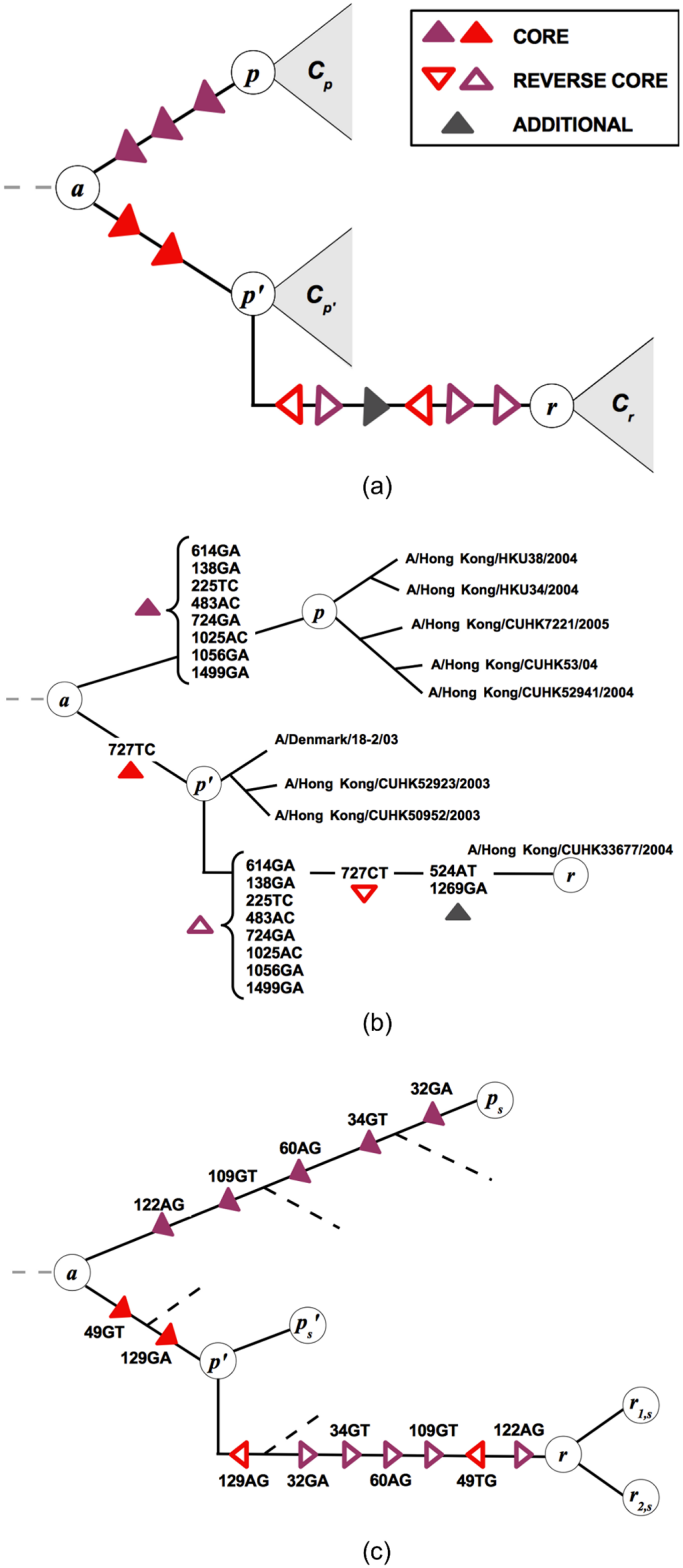
Tree representation of reassortment. (a) Representation of reassortment in a two-segment genealogical tree. The parent strains *p* and *p*′ are in different sublineages of the tree; the reassortant strain *r* appears as a descendant of one of these parents (here *p*′; there is an equivalent tree in which *r* appears as a descendant of *p*). The strains *p*, *p*′, and *r* are the focal nodes of the clades *C*_*p*_, *C*_*p*′_, and *C*_*r*_, respectively (grey areas). We identify the reassortment event by its set of core mutations, $A_{pp^{\prime }}$, which appear on the segment that *r* inherits from *p* and generate the genetic distance between the parent strains in that segment. The core mutations appear on the branches between the nodes *p* and *p*′ (filled red triangles: mutations between *p* and the last common ancestor *a*, filled purple triangles: mutations between *a* and *p*′). Their reverse mutations appear on the branch between *p*′ and *r* (empty red and purple triangles), which can also contain additional mutations (grey triangles). (b) A true event (nr 1 in S2 Table) detected by our algorithm on the joint HA-NA tree. Each mutation on HA segment is labeled with a number between 1 and 1701 that indicates the site. The pattern of repeated and reversed mutations (filled and empty triangles) follows the scheme in Fig. 2a: the reassortant strain A/Hong Kong/CUHK33677/2004 is generated by an event with *δ* = 9 between *p* and *p*′ clades. (c) The result of a simulated reassortment event on the reconstructed genealogical tree, correctly detected by the algorithm. The internal node *r* is inferred as the reassortant ancestor of *r*_1/2,*s*_, i.e. the strains evolved from the sequence that was actually generated by reassortment between *p*_*s*_ and $p_{s}^{\prime }$.

A reassortant strain reassembles the genome segments of two parent strains that co-infect a host cell. In this study, we focus on reassortment between the HA and NA genes of influenza, because the evolution of both proteins has been linked to immune escape and functional epistasis between them affects vaccine efficacy [40, 41]. Hence, we restrict the genomic analysis to the two segments carrying the HA and NA genes; each parent strain contributes exactly one of these segments to the reassortant strain (Fig 1). Our inference of reassortment is based on genealogical trees constructed from linked sequence of these two segments (Materials and methods). A tree representation of a joint genealogy with a reassortment process is shown in Fig 2a. The two parental strains *p* and *p*′ appear in different sublineages, and the reassortant strain *r* is shown as a descendant of one of these parents (here *p*′). These strains define three distinct clades of descendant strains, *C*_*p*_, *C*_*p*′_, and *C*_*r*_ (grey areas in Fig 2a); the numbers of strains in these clades are denoted by *n*_*p*_, *n*_*p*′_, and *n*_*r*_, respectively. We note that the “direction” of the reassortment event (here from *p* to *p*′) is merely a property of the tree representation, and there is an equivalent tree with the roles of *p* and *p*′ exchanged. This reassortment pattern can be readily identified in two-segment trees. Fig 2b shows an example of a HA-NA reassortment event in the genealogy of influenza A/H3N2, another example using tree data from simulated evolution of a population in a regime of clonal interference is shown in Fig 2c.

**Fig 3:**
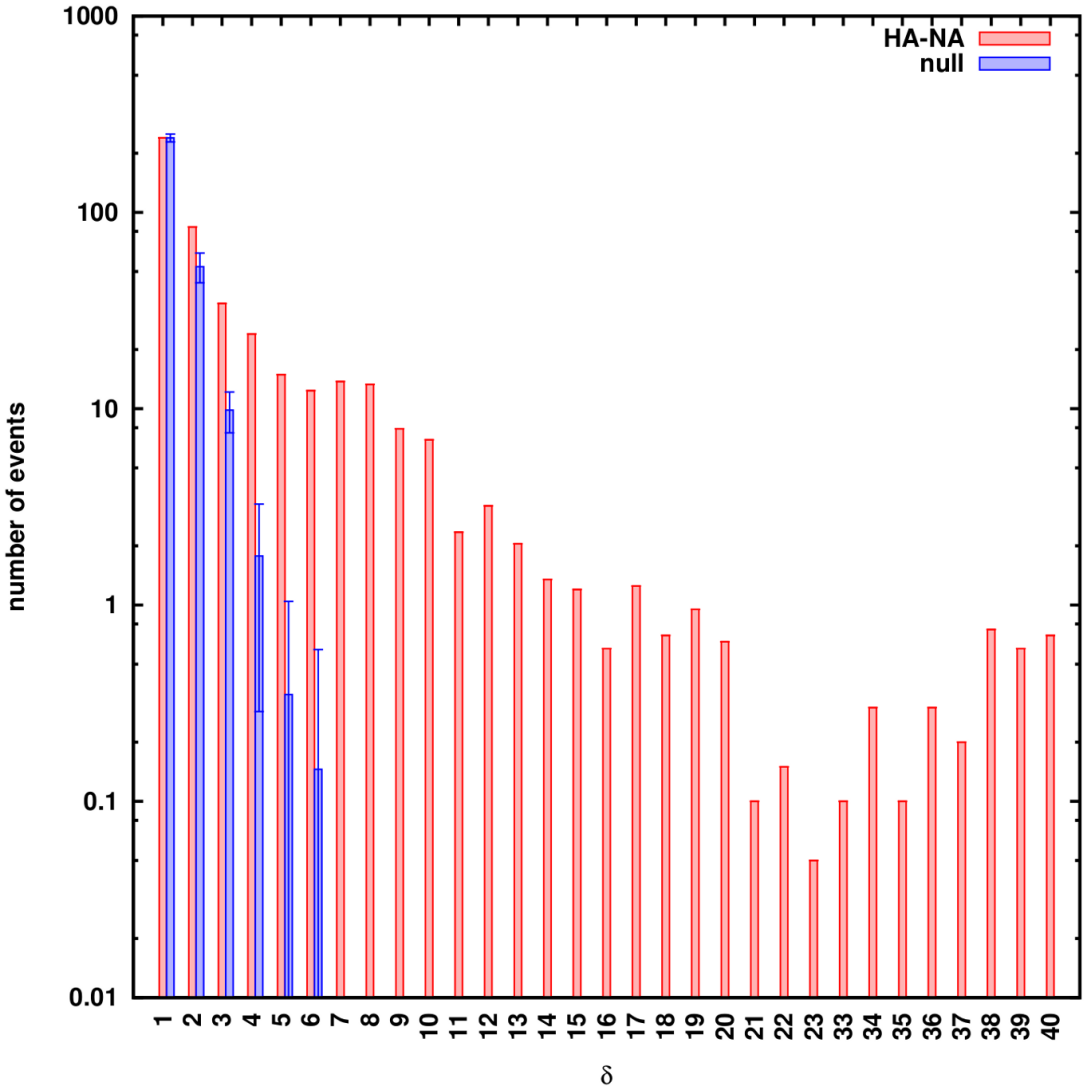
Fidelity of reassortment inference. Histograms of reported HA-NA reassortment events for different core distances *δ* (red bars) are compared to expected number of false positives due to ambiguities in tree reconstruction, *n*_0_(*δ*), from a null model of non-reassorting sequences (blue bars; error bars reflect the statistics over different realizations of the null model). The function *n*_0_(*δ*) decreases exponentially with increasing *δ* (cf. Eq 6 in Materials and methods); the overall amplitude is set by the conservative assumption that all counts at *δ* = 1 are false positives. The resulting total number of false positives with *δ* ≥ 5 is below 1.

We identify candidate reassortment events (*p*, *p*′, *r*) from their signal in a two-segment genealogical tree: a set of core mutations, $A_{pp^{\prime }}$, appears on the branches between the nodes *p* and *p*′, and their reverse mutations appear on the branch between *p*′ and *r*. These mutations are in the segment that *r* inherits from *p* (Fig 2a). The resulting list of events must undergo further statistical analysis: false positives must be excluded and candidates representing the same reassortment event must be clustered. In Materials and Methods, we detail our inference scheme and show that its fidelity strongly depends on the number of core mutations, $\delta =|A_{pp^{\prime }}|$. Inferred events with *δ* ≥ 5 are very likely to be true reassortments, while events with *δ* ≤ 4 are enriched with false positive counts reflecting alignment ambiguities (Fig 3).

To characterize the span of a reassortment event, we use the mean genetic distance between the parent strains *p* and *p*′ in both segments, $d=\frac{1}{2}\left(d_{\text{HA}}+d_{\text{NA}}\right)$ (we evaluate these distances for nucleotides and for amino acids). The quantity *d* is also the mean genetic distance of the reassortant strain *r* from its parents; below, we report evidence for negative epistasis between these mutations.

### Rate and genealogy of reassortment for influenza A/H3N2

We apply our reassortment inference method to a sequence dataset of human influenza A/H3N2 collected from 1968 to 2015. In each two-segment tree, we map HA-NA reassortments as detailed in Methods: we identify candidate events (*p*, *p*′, *r*) by the criterion (Eq 5), we keep only events with core distance *δ* ≥ 5, we prune events with strongly overlapping core sets $A_{pp^{\prime }}$, and we eliminate double-counting of events. At the same time, the total number of detected events does not depend on the number of inferred trees, indicating that our mapping exhausts the events occurring in the original dataset. Furthermore, we have verified that passaging mutations do not confound our inference (Methods).

This procedure produces a list of 103 bona fide reliable and independent HA-NA reassortments in our data set. These events have a mean genetic distance *d* ≥ 3 between reassortant and parent strains in both segments and an average *d*_ave_ = 10, which sets them clearly apart from individual point mutations and provides the genetic basis for potentially strong and epistatic selection (see below). In S2 Table, we report the genetic distance *d*, as well as representative strains from the reassortant clade *C*_*r*_ and the parent clades *C*_*p*_, *C*_*p*′_. From the year 2000 on, we find an average of 6 unique reassortment events per year, which is in overall agreement with other studies [37–39]. Furthermore, events detected by the majority of these studies are well represented in our list (starred events in S2 Table); these include reassortments between New York strains isolated between 2000 and 2005 [13, 28, 32, 34] (see S1 Text for more details). The clean statistical test used here, however, addresses the over-counting of events in a more objective way.

**Fig 4:**
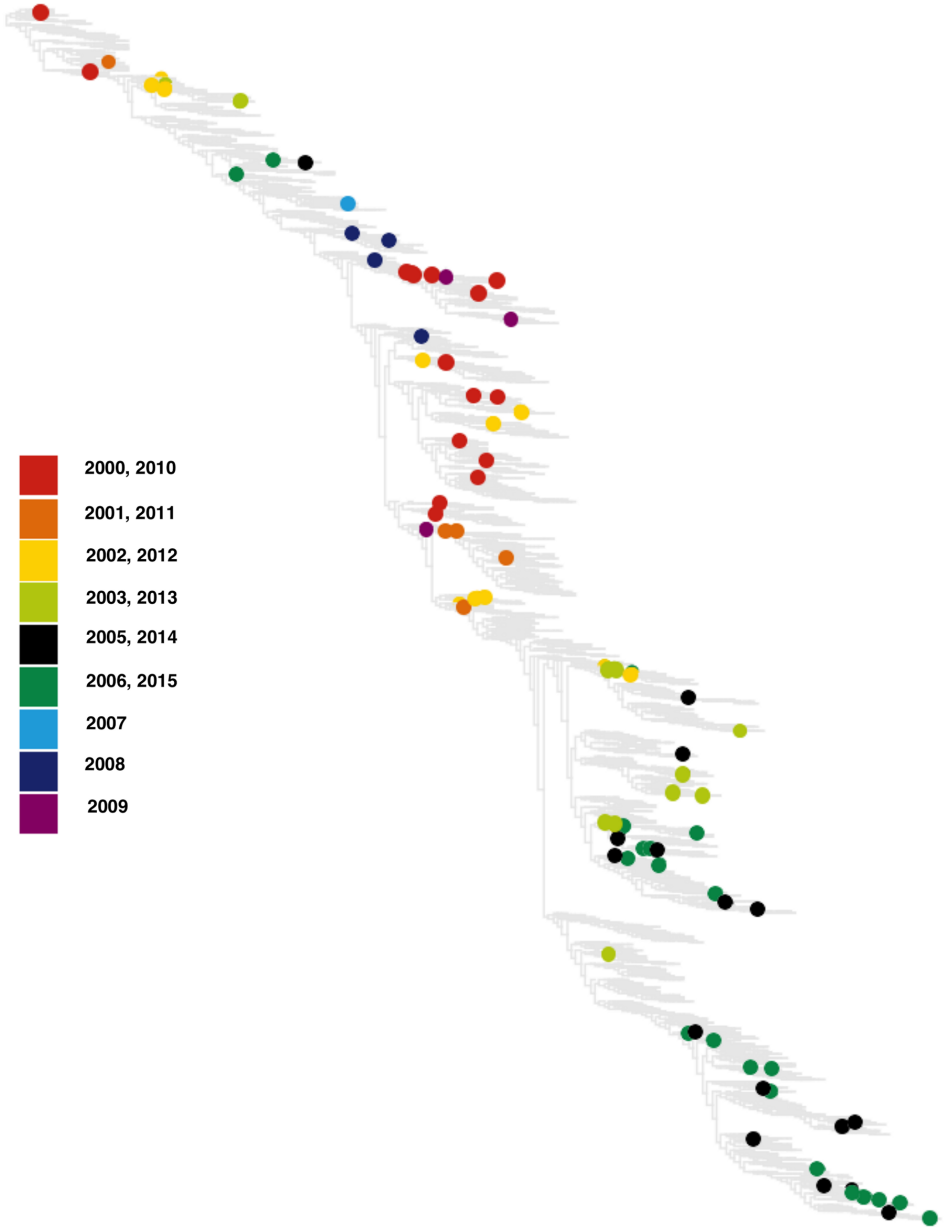
Reassortment of HA and NA in human influenza A/H3N2 from 2000 to 2015. The 95 inferred events are mapped on a joint HA-NA tree. The reassortant strain *r* of each event is represented by a filled circle (color-coded by year of occurrence). The events are homogeneously distributed over the tree and the reassortant clades are predominantly at peripheral positions of the tree.

Fig 4 shows the inferred reassortments since 2000 mapped on a joint HA-NA tree. These events cover the entire time interval of the tree with a slight increase in frequency in recent years, which is likely due to increased depth of the tree. In all cases, the parent strains were collected at close times, which is consistent with the fast evolutionary speed and the resulting short sojourn periods of specific genotypes in the population of circulating strains. By comparing the number of reassortment events with the number of synonymous nucleotide changes on the same tree, we estimate that reassortant variants get established at a rate of order 10^−2^ in units of the neutral point mutation rate. This establishment rate refers to observed variants in a strain sample, which clearly depends on the sampling depth (our data set has a detection threshold frequency of order 10^−3^). Our finding of broad negative selection on reassortment, which is reported below, suggests that the reassortment rate of individual virions is higher, but many reassortant variants are rapidly lost in the population of circulating strains.

### Reassortment is under broad negative selection

As shown in Fig 4, the majority of observed reassortment events are on peripheral positions of the joint HA-NA tree. This observation is broadly consistent with a neutral or, on average, deleterious process. We now turn to measuring selection on reassortment in a more quantitative way.

**Fig 5:**
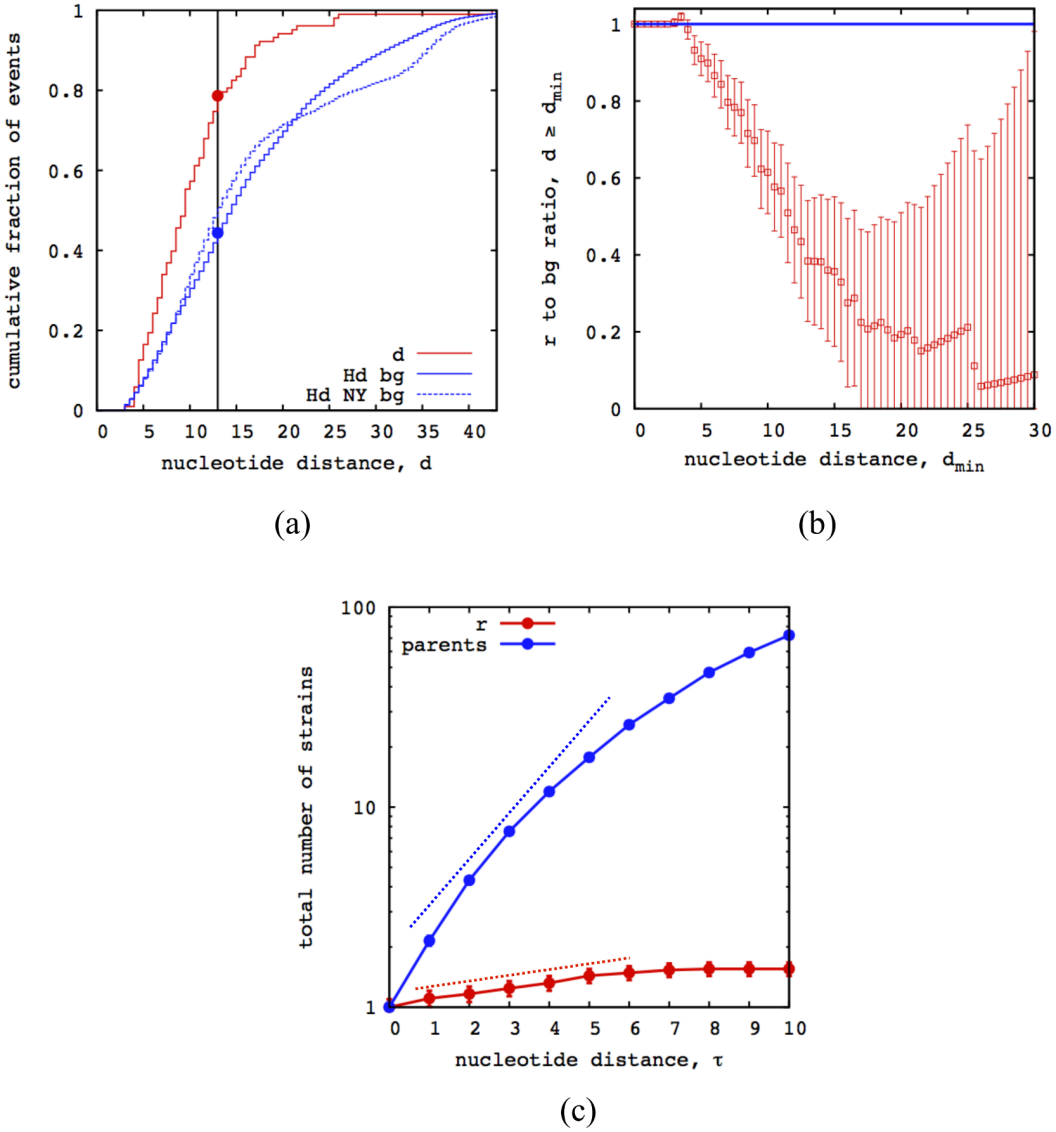
Negative selection on reassortment. (a) The cumulative distribution of mean nucleotide distances *d* between reassortant and parent strains for the HA-NA reassortments in influenza A/H3N2 (red line) is compared to the corresponding distribution of distances for co-circulating strains in the same influenza season (solid blue line) and from the New York area only [28] (dashed blue line). (b) The ratio of reassortment counts to background counts in the interval *d* ≥ *d*_min_ (red circles) decreases with increasing lower threshold *d*_min_ and drops significantly below 1 (blue line). The suppression of reassortment at larger values of *d* signals distance-dependent negative selection. Bars show statistical errors due to the finite number of inferred reassortments. (c) The average number of strains in the reassortant clades with nucleotide distance ≤ *τ* from the focal node, 〈*N*_*r*_〉(*τ*) (red line) is compared to the corresponding average number of strains in the parent clades, 〈*N*_0_〉(*τ*). For *τ* ≲ 6, both functions increase with *τ* in an approximately exponential way; we estimate growth rates *f*_*r*_ ≈ 0.07 and *f*_0_ ≈ 0.5, respectively (dashed lines; cf. Eq 1). The growth rate difference $\bar{s}\equiv f_{0}-f_{r}\approx 0.4$ measures the average fitness cost of reassortment. Bars represent statistical errors due to the finite number of counts (not shown when these errors are smaller than the dot size). See S3 Fig for an analogous inference based on amino acid distances.

First, we compare the distribution of distances in detected reassortment events, *P*(*d*), with the corresponding background distribution sequence distances between all pairs of strains circulating in a given influenza season, *P*_0_(*d*) (both distributions are defined in the regime *d* ≥ 3). The latter distribution represents the background pool of a priori equiprobable opportunities for reassortment. In the absence of selection, reassortment should occur with equal probability between these pairs, regardless of their genetic distance, and the distribution *P*(*d*) should be similar to the background distribution *P*_0_(*d*). However, Fig 5a shows significant differences between these distributions: there are far fewer actual events with larger values of *d* than in the background distribution. We measure the statistical significance of these differences by the Kullback-Leibler (KL) divergence *D*_*KL*_ = ∑_*d*≥3_ *P*_0_(*d*) log(*P*_0_(*d*)/*P*(*d*)) and by the Kolmogorov-Smirnov statistics, *D*_*KS*_ = max |*F*(*d*) − *F*_0_(*d*)|, where *F*(*d*) and *F*_0_(*d*) are the corresponding cumulative distributions. We find the suppression of high-*d* reassortment events to be significant by both tests, with *D*_KL_ = 0.56 (compared to a 5% error threshold at *D*_KL_ = 0.1) and *D*_KS_ = 0.34 (giving a probability *p* < 10^−23^ to find a larger distance by chance). As shown by Fig 5b, the ratio of reassortment to background counts with *d* ≥ *d*_min_ decreases as a function of the lower cutoff *d*_min_. We attribute this effect to distance dependent deviations from neutrality: negative selection on reassortment increases in strength with distance *d*. The same analysis based on amino acid distances, which provide a more coarse-grained measure of genetic differences, also shows a significant suppression of large-*d* reassortment events (S3 Fig).

A potential confounding factor for this analysis is the spatio-temporal population dynamics of the human influenza virus [12, 42]. Although influenza strains are known to travel rapidly, the local background distribution *P*_0_ of genetic distances, which matters for reassortment, can in principle differ from its global counterpart used in our significance analysis. In order to estimate this effect, we restrict the background distribution to strains that circulate in the same region; specifically, we calculate an alternative distribution *P*_0_ using isolates from New York State only, which are available from a previous study focusing on that region [28] (as before, Hamming distances are computed only between strains reported in the same influenza season). We still find that the actual reassortment events differ significantly from the local distribution *P*_0_ (Fig 5a and S3 Fig), while the global and the local background distributions are statistically indistinguishable. We conclude that the suppression of large-*d* reassortment is not a spurious demographic effect, but is indicative of selection.

To gain some insight on how reassortment constraint is distributed on the amino acid distances in individual segments, *d*_HA_ and *d*_NA_, we evaluate the joint background distribution *P*_0_(*d*_HA_, *d*_NA_) and compare it with the amino acid distance pairs (*d*_HA_, *d*_NA_) of the inferred reassortment events (S4 Fig). The joint statistics of (*d*_HA_, *d*_NA_) differs in the coordinates *d*_HA_ + *d*_NA_ and *d*_HA_ − *d*_NA_, indicating that selection is not a function of *d* only. In particular, the conditional distributions *P*(*d*_HA_ − *d*_NA_|*d*) differ between data and background (S4 Fig), which is consistent with the expectation that reassortants similar in one protein are less selected against, even if the distance in the other protein is larger.

To quantify negative selection on reassortment directly within the set of observed events, we compare the evolution of population sizes of reassortant clades and of parent clades as a function of their age *τ*. We evaluate, for each reassortment event, the number *N*_*r*_(*τ*) of strains in the reassortant clade *C*_*r*_ with nucleotide distance ≤ *τ* from the focal node *r*, together with the mean of the corresponding numbers of strains in the parent clades, *N*_0_(*τ*) = (*N*_*p*_(*τ*) + *N*_*p*′_(*τ*))/2. We obtain these functions in the joint HA-NA tree of the full data set, counting strains with the same sequence only once. Averaging over the set of reassortment events, we can measure the expected growth of reassortant and parent clades,

$$\left\langle N_{r}\right\rangle \left(\tau \right)∼\text{exp}\left(f_{r}\tau \right),\;\left\langle N_{0}\right\rangle \left(\tau \right)∼\text{exp}\left(f_{0}\tau \right);$$ (1)

similar inference methods for clade growth are discussed in refs. [43, 44]. The functions 〈*N*_*r*_〉(*τ*) and 〈*N*_0_〉(*τ*) for influenza A/H3N2 indeed show approximately exponential growth in the regime *τ* ≲ 6, which corresponds to time intervals of order one year (Fig 5c). The fitted growth rate difference estimates the average fitness cost of reassortment in our set of events,

$$\begin{array}{c} \bar{s}\equiv f_{0}-f_{r}\approx 0.4 \end{array}$$ (2)

in units of the total point mutation rate in both segments, which equals approximately 5 × 10^−3^ per day. The same analysis performed with aminoacid distances is reported in S3 Fig.

### Epistasis across proteins

To further interpret the observed fitness cost of reassortment, we consider the simplest epistatic fitness model for combined (HA, NA) genotypes, *F*_*αβ*_ ≡ *f*(HA_*α*_, NA_*β*_), where *α*, *β* = +1 denote the alleles of the parent strain *p* and *α*, *β* = −1 the alleles of parent strain *p*′. The model takes the form

$$\begin{array}{c} F_{\alpha \beta }=f_{\alpha }^{\text{HA}}+f_{\beta }^{\text{NA}}+\frac{\omega }{2}\alpha \beta , \end{array}$$ (3)

where *f*^HA^ and *f*^NA^ denote single-protein fitness values and *ω* is the strength of cross-protein epistasis. In terms of this model, the mean fitness cost of a reassortant strain compared to its parent strains is

$$\begin{array}{c} s=\frac{1}{2}\left(f_{p}+f_{p^{\prime }}\right)-f_{r}=-\frac{1}{2}\left(f_{+}^{\text{HA}}-f_{-}^{\text{HA}}\right)-\frac{1}{2}\left(f_{-}^{\text{NA}}-f_{+}^{\text{NA}}\right)+\omega , \end{array}$$ (4)

where we assume, without loss of generality, that the reassortant strain *r* inherits HA from parent *p* and NA from parent *p*′. If co-infection randomly mixes co-circulating strains, the single-protein fitness value of a reassortant strain is, on average, equal to the mean fitness of its parents. For strains observed in a sequence sample, this value can only be biased towards larger reassortant fitness; i.e., $\frac{1}{2}\langle f_{+}^{\text{HA}}-f_{-}^{\text{HA}}\rangle \geq 0$ and $\frac{1}{2}\langle f_{-}^{\text{NA}}-f_{+}^{\text{NA}}\rangle \geq 0$. Hence, the observed fitness cost (2) implies an average epistatic cost of reassortment, $\langle \omega \rangle >\bar{s}>0$. Cross-protein epistasis in the observed reassortment events is of moderate strength but broadly distributed: reassortant variants are fit enough to reach population frequencies detectable in our sample, but they are, on average, less fit than their non-reassortant counterparts.

## Discussion

We have developed a new method to map reassortment of genomic segments in an evolving viral population. We detect reassortment events based on their trace in the genealogy of the population. On a two-segment genealogical tree, a set of *core mutations* in one of the reassorted proteins appears twice: on the branches linking the reassorting (parent) strains and, in reverse direction, on the branch to the reassortant clade (Fig 2a). We are interested predominantly in reassortment events above a certain minimum genetic distance *d* from their parent strains (here at least 3 mutations), which are clearly set apart from the dynamics of point mutations. This is also the regime in which our method allows a reliable identification of events, which is not confounded by ambiguities in tree reconstruction (Fig 3).

The main biological result of this paper is that reassortment within human influenza A/H3N2 is under broad, distance-dependent negative selection. Specifically, there are fewer large-*d* reassortments in our sample than expected from the distribution of co-circulating strains, and reassortant strains have fewer descendants than their parent strains (Fig 5). These observations probe negative selection on reassortant genotypes at different scales of frequency and sojourn time in the population. The suppression of large-*d* reassortment signals purifying selection that prevents some reassortant variants from reaching sufficient frequencies to appear in our strain sample; the growth rate difference between reassortant and parent clades indicates moderate negative selection on the variants that do appear in the sample. Reassortment between very close sequences may well be approximately neutral, but sequence-based inference methods cannot distinguish such events reliably from point mutations. The inferred selective effects characterize the continuous evolution of a seasonal influenza lineage; they do not exclude rare large-effect reassortment events causing antigenic shifts and seeding new lineages. We stress that our results are based on statistical methods evaluating ensembles of inferred reassortments and background distributions. Any such method is subject to possible confounding factors and biases; for example, reassortment can be expected to occur preferentially in high-infection settings at the peak of seasonal epidemics. It is the consistent outcome of two distinct inference procedures that gives a credible signal of selection acting on reassortment.

Reassortment can be seen as a natural “experiment” that continuously produces new combinations of viral proteins and probes their fitness in a fast-evolving population. Hence, the statistics of reassortment is informative of key selective forces governing the evolutionary dynamics. Specifically, our result of negative selection on reassortment signals ubiquitous fitness interactions (epistasis) between viral proteins; that is, the fitness of alleles of one protein depends on the genetic background of the other proteins in the same virion. The mutation-selection dynamics in non-reassortant sublineages produces favorable combinations of protein alleles, and reassortment introduces a fitness cost by randomizing these combinations. Importantly, this cost arises between genetic variants from co-circulating strains in a given viral lineage; these variants are individually viable, differ by just a few mutations (of order 20 nucleotide changes in both reassorted segments together), and have a recent common ancestor (typically dating just one or two years back). This implies that new favorable protein combinations are continuously produced and selected for, while many random combinations incur a fitness cost. In other words, cross-protein epistasis constrains the adaptive evolutionary path of a given influenza lineage. This result could be tested, for example, by combining reassortment experiments [16–22] with in-vitro competitive fitness assays. Reassortant strains with substantial distances *d* should frequently be outcompeted by any of their parent strains.

The resulting negative selection on reassortment increases in strength with the genetic distance between reassortant and parent strains (Fig 5b). The mixing of genetic material by reassortment is somewhat similar to recombination in diploid populations or transformation in bacteria. However, recombination and transformation require physical splicing of genome segments; the rates of these enzymatic processes get strongly suppressed with increasing genetic distance of the parent sequences [45–47]. There are no corresponding physiological barriers against reassortment of viral segments. Instead, our results suggest that epistatic fitness barriers are already substantial between more distant co-circulating strains of the same lineage, which ties in with the observation of cross-lineage pairing constraints [22, 24]. Hence, on larger scales of genetic distance, such fitness barriers may be an important factor in delineating—and, thus, defining—viral species.

## Materials and methods

### Alignments and genealogical trees

A sample of HA and NA sequences is obtained by downloading all the available A/H3N2 human strains in the EpiFlu DATABASE (http://www.gisaid.org), regardless the geographical region, which were collected between January 1968 and October 2015. Only the strains with complete HA and NA sequences are taken into consideration. Acknowledgements for sequences used in this study are available in S1 File. From this sample, alignments of single segments are created year by year using BLAST [48]. After discarding the segments with more than three gaps, a first run of RAxML [49] is performed to reconstruct the genealogy for each segment and detect clear outliers sequences (i.e. sequences that are found clearly isolated from the rest of the tree and which were likely misreported) to be excluded from the subsequent analysis. We then obtain alignments of linked HA-NA segment pairs and construct maximum-likelihood two-segment genealogies by RAxML, choosing the best-scoring ML tree out of 10 RAxML runs. Potential ambiguities in the definition of the nucleotide at a certain site in a strain are resolved by assigning the orthologous nucleotide of the closest ancestral node with an unambiguous sequence. The subsequent reassortment inference is performed on trees of subsampled data with a maximum of 600 sequences per year. This step reduces computational time and avoids over-representation of recent viruses, which are the most abundant in the database. We note that these joint genealogies differ from single-segment phylogenetic trees, because the underlying process of reassortment violates the tree topology.

Some of the isolates we use in this study were subject to passaging for amplification in cell culture. To test the possible role of passaging adaptations as a confounding factor [50], we repeat our inference of reassortment on 20 trees built from a restricted alignment of 1053 unpassaged sequences. We observe the same patterns of mutations which characterize the reassortment events reported above, as well as a very similar distance dependence of the false positives counts (S2 Fig). The robustness of our inference is expected, because we only consider events with mean genetic distance *d* ≥ 3 between reassortant and parent strains in the reassorting segments.

### Primary inference of reassortment events

Consider the tree representation of a reassortment event with parental strains *p* and *p*′ and the reassortant strain *r* (Fig 2a). This representation defines one of the two segments, referred to as the travelling segment, and a set of mutations which generate the genetic distance between the parent strains *p* and *p*′ in that segment. These so-called core mutations appear on the branches between the nodes *p* and *p*′, which pass through their last common ancestor *a*. We define the set $A_{pp^{\prime }}$ of core mutations by counting the mutations from *p* to *a* in upward direction on the tree (filled red triangles) and the mutations from *a* to *p*′ in downward direction (filled purple triangles). The branch from *p*′ to *r* contains a set of mutations $A_{p^{\prime }r}$ that includes the set of reverse core mutations, denoted by ${\bar{A}}_{pp^{\prime }}$ (open red and purple triangles), as well as additional mutations in the travelling segment (grey triangles). These mutations reflect insufficient sampling (i.e., one of the actual parent strains is not included in the tree) and noise in the reconstruction of the phylogeny (see below), or they are point mutations unrelated to the reassortment event. Together, we obtain a criterion to detect reassortment in a two-segment tree: we parse the tree for node triplets (*p*, *p*′, *r*) with

$$\begin{array}{c} A_{p^{\prime }r}⊇{\bar{A}}_{pp^{\prime }} \end{array}$$ (5)

in a given travelling segment.

### Pruning steps: Uniqueness and false positives

The detection of reassortment on genealogical trees overcounts the biological reassortment events. First, we exclude false positive events due to ambiguities in tree reconstruction by statistical comparison with a null model. Since recombination within segments does not occur in influenza, we use as null case a set of trees built from the alignments of the single segments. We decompose the sequence of the protein into two subsets of randomly chosen sites with lengths *L*_1_ and *L*_2_. These subsets have the appropriate ratio of lengths to mimic the segment structure in the original joint alignment, *L*_1_/*L*_2_ = *L*_HA_/*L*_NA_. In order to investigate the dependence of the number of false positive reassortments on the length of the chain, we run the detection algorithm on subsets of sites of increasing total length *L* = *L*_1_ + *L*_2_, maintaining a constant ratio *L*_1_/*L*_2_. We find an expected number *n*_0_(*δ*) of false positive reassortment events that decays rapidly with increasing core distance,

$$\begin{array}{c} n_{0}\left(\delta \right)=Ce^{-\gamma \delta } \end{array}$$ (6)

with *γ* = 1.6 ± 0.1. The decay exponent *γ* is approximately independent of the total sequence length *L* (S1 Fig). We can then evaluate the expected number of false positive reassortment events in the actual data (Fig 3 and S1 Table). Even if we assume that most of the counts at *δ* = 1 are false positives (which sets the value of the constant *C* in Eq 6); the expected total number of false positive events with *δ* ≥ 5 drops below 1 (Fig 3 and S1 Table).

Second, two or more different reassortment events reported by our algorithm may represent the same biological reassortment event if they have similar core set. To address this source of overcounting, we compare the core sets $A_{pp^{\prime }}$ of putative events. If these sets differ by at least 30% of their mutations, the events are considered independent; otherwise we keep only the set with the largest core distance *δ*. The number of pruned events turns out to be insensitive to moderate changes of the threshold number of mutations. As third step, we cluster the reported events with different travelling segments that have very similar parent strains.

### Testing the inference method by simulations

We simulate the genome evolution of a population of *N* individuals starting at a stationary state, under the effect of mutation, genetic drift and selection, based on the model used in [5]. Each strain is characterized by a sequence of epitope and non-epitope sites, flanked by neutral sites. Selection on epitope sites is time-dependent and its direction fluctuates randomly at a rate *γ*, while non-epitope sites are modeled with time-independent direction. To these basic steps we add reassortment, which occurs at each generation with probability λ: we select randomly two individuals (the parents) and divide their genome into two parts of fixed length *L*_1_ and *L*_2_, then mimic the process of reassortment by creating a new individual (*r*) with a mixed genome. We focus on events between strains at genetic distance *d*_1,2_ ≥ 5 in each segment, discarding reassortment at lower distances. The results of each simulation are a set of sampled sequences, some of them involved in a reassortment event, that we use to build up the genealogical trees, as we would do with real observed strains.

We choose the parameters of the simulations as follows:

1. The evolution of *N* = 1000 individuals is simulated for 1500 generations. Each individual has a genome of length *L* = *L*_*ep*_+*L*_*non*−*ep*_+*L*_*neut*_ = 560 (*L*_*ep*_ = 120, *L*_*non*−*ep*_ = 160, *L*_*neut*_ = 300 number of epitope, non epitope and neutral sites, respectively), selection flips the direction at rate *γ* = 0.033 and the mutation rate is set to *μ* = 5.8 × 10^−3^ per year. With these evolution parameters, the population turns out to be in a clonal interference regime comparable to influenza [5].
2. We introduce reassortment at a rate rate λ = 1 × 10^−6^ per individual and per generation. This generates a density of reassortant variants at observable population frequencies that is comparable with the observed density in influenza A/H3N2.
  With these parameters, we obtain trees that show ∼ 5 coalescent events on average, corresponding to approximately 10 years of influenza evolution. We apply our algorithm on each of the 100 reconstructed trees and check if the reassortment events recognizable in the sampled sequences (i.e. the ones with *r* and/or its offspring reaching a relevant frequency and therefore getting sampled) get detected. Out of the total 283 events generated in the simulations, 214 (76%) are correctly reported (see Fig 2c for an explicit example of a detected event), with 24 false positives signaled with small cores (*δ* ≤ 5).

## Supporting information

S1 Fig Distance dependence of spurious reassortment counts in non reassorting sequence. (a) Histograms of the number of events found in a HA tree as a function of *δ*, for sequences of total length *L*. Error bars represent the standard deviation obtained from 5 different random choices of the sites for each *δ*. (b) The decay exponent *γ* is shown as a function of *L* (cf. Materials and methods, Eq 6). The inferred values are stable for large values of *L*, allowing extrapolation to *L* = *L*_HA_ + *L*_NA_.
(PDF)

S2 Fig Reassortment inference between unpassaged sequences. Histograms of reported HA-NA reassortment events between unpassaged sequences for different core distances *δ* (red bars) are compared to expected number of false positives (blue bars), which decays exponentially with increasing *δ*. This result is qualitatively comparable with the distance dependence of real events and false positives that we find including in the analyses also strains subjected to passaging.
(PDF)

S3 FigSelection inference based on aminoacid distances.(a) The cumulative distribution of mean amino acid distances *d* between reassortant and parent strains for the HA-NA reassortments in influenza A/H3N2 (red line) is compared to the corresponding distribution of distances for co-circulating strains in the same influenza season (solid blue line) and from the New York area only (dashed blue line). (b) The ratio of reassortment counts to background counts in the interval *d* ≥ *d*_min_ (red circles) decreases with increasing lower threshold *d*_min_ and drops significantly below 1 (blue line). The suppression of reassortment at larger values of *d* signals distance-dependent negative selection. Bars show statistical errors due to the finite number of inferred reassortments. See Fig 5 for the same analysis using nucleotide distances. (c) The average number of strains in the reassortant clades with aminoacid distance ≤ *τ*_*A*_ from the focal node, 〈*N*_*r*_〉(*τ*_*A*_) (red line) is compared to the corresponding average number of strains in the parent clades, 〈*N*_0_〉(*τ*_*A*_). For *τ*_*A*_ ≲ 4, both functions increase with *τ*_*A*_ in an approximately exponential way; we estimate growth rates *f*_*r*_(*A*) ≈ 0.2 and *f*_0_(*A*) ≈ 0.7, respectively (dashed lines; cf. Eq 1). The growth rate difference ${\bar{s}}_{A}\equiv f_{0}\left(A\right)-f_{r}\left(A\right)\approx 0.5$ inferred from distances in aminoacid units is similar to $\bar{s}\approx 0.4$ for nucleotide distances; cf. Fig 5c.(PDF)

S4 FigBackground distribution and reassortment events as a function of the amino acid distances *d*_HA_ and *d*_NA_ between strains.(a) The background distribution $P_{0}^{aa}\left(d_{\text{HA}},d_{\text{NA}}\right)$ (contour plot) is compared to reassortment counts (red dots). (b) Conditional background distributions $P_{0}^{aa}\left(d_{\text{HA}}-d_{\text{NA}}|d_{\text{HA}}+d_{\text{NA}}\right)$ (whisker plots) are compared to reassortment counts (red dots). Whisker plots show the 0.25 quantile to the 0.75 quantile of the distribution (blue boxes); the white horizontal line represents the median, vertical bars span the dataset excluding outliers. The width of the bins is chosen to ensure a statistically relevant number of events for each bin. The reassortment data appear more spread in the coordinate *d*_HA_ − *d*_NA_ compared to the background (red points are mainly placed outside or at the border of the blue boxes).(PDF)

S1 Table Number of expected false positive reassortment counts as a function of *δ* (cf. Fig 3). (PDF)

S2 Table List of inferred reassortment events from 1968 to 2015 between HA and NA segments in human influenza A/H3N2. Column 2: mean nucleotide distance *d* between reassortant strain and parent strains. Columns 3–5: representative observed strains in the clades of *p*, *p*′ and *r*, respectively. Each isolate is identified by its number in the online EpiFlu DATABASE (http://www.gisaid.org) identifier (e.g. EPI_ISL_7064 is reported here as 7064). Stars indicate events which are reported in literature with large agreement (S1 Text).
(PDF)

S1 Text Comparison with reassortment reported in literature. (PDF)

S1 File GISAID acknowledgement table. (XLS)

S1 Code Compressed code folder. (ZIP)
