# Supplementary material for: Fitness cost of reassortment in human influenza
Source: PLoS Pathog. 2017 Nov 7;13(11):e1006685. doi: 10.1371/journal.ppat.1006685 (PMC5675378; doi:10.1371/journal.ppat.1006685)
Supplement: S1 Fig — (a) Histograms of the number of events found in a HA tree as a function of δ, for sequences of total length L. Error bars represent the standard deviation obtained from 5 different random choices of the sites for each δ. (b) The decay exponent γ is shown as a function of L (cf. Materials and methods, Eq 6). The inferred values are stable for large values of L, allowing extrapolation to L = LHA + LNA. (PDF) [file ppat.1006685.s001.pdf]

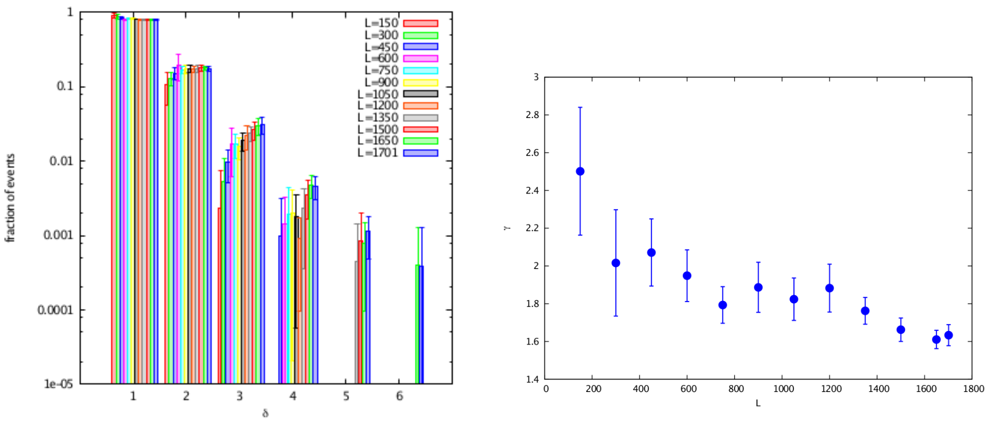

(a) (b)

S1 Fig. Distance dependence of spurious reassortment counts in non reassorting sequence. (a) Histograms of the number of events found in a HA tree as a function of δ, for sequences of total length L. Error bars represent the standard deviation obtained from 5 different random choices of the sites for each δ. (b) The decay exponent γ is shown as a function of L (cf. Materials and Methods, equation 6). The inferred values are stable for large values of L, allowing extrapolation to $L = L_{\mathrm{HA}} + L_{\mathrm{NA}}$.
