# Supplementary material for: Fitness cost of reassortment in human influenza
Source: PLoS Pathog. 2017 Nov 7;13(11):e1006685. doi: 10.1371/journal.ppat.1006685 (PMC5675378; doi:10.1371/journal.ppat.1006685)
Supplement: S2 Fig — Histograms of reported HA-NA reassortment events between unpassaged sequences for different core distances δ (red bars) are compared to expected number of false positives (blue bars), which decays exponentially with increasing δ. This result is qualitatively comparable with the distance dependence of real events and false positives that we find including in the analyses also strains subjected to passaging. (PDF) [file ppat.1006685.s002.pdf]

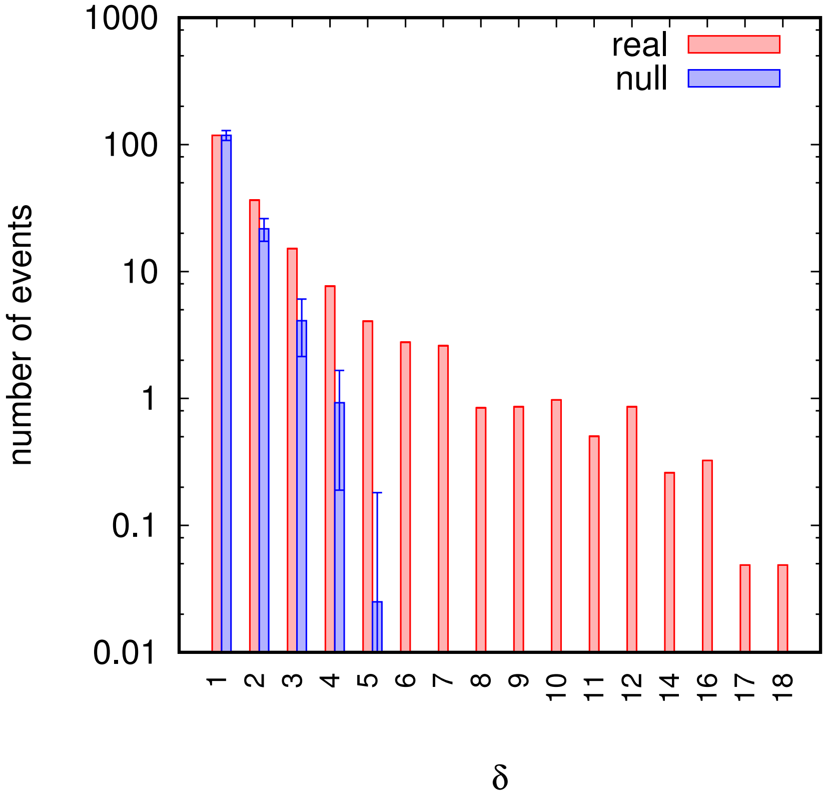

S2 Fig. Reassortment inference between unpassaged sequences. Histograms of reported HA-NA reassortment events between unpassaged sequences for different core distances δ (red bars) are compared to expected number of false positives (blue bars), which decays exponentially with increasing δ. This result is qualitatively comparable with the distance dependence of real events and false positives that we find including in the analyses also strains subjected to passaging.
