# Supplementary material for: Fitness cost of reassortment in human influenza
Source: PLoS Pathog. 2017 Nov 7;13(11):e1006685. doi: 10.1371/journal.ppat.1006685 (PMC5675378; doi:10.1371/journal.ppat.1006685)
Supplement: S3 Fig — (a) The cumulative distribution of mean amino acid distances d between reassortant and parent strains for the HA-NA reassortments in influenza A/H3N2 (red line) is compared to the corresponding distribution of distances for co-circulating strains in the same influenza season (solid blue line) and from the New York area only (dashed blue line). (b) The ratio of reassortment counts to background counts in the interval d ≥ dmin (red circles) decreases with increasing lower threshold dmin and drops significantly below 1 (blue line). The suppression of reassortment at larger values of d signals distance-dependent negative selection. Bars show statistical errors due to the finite number of inferred reassortments. See Fig 5 for the same analysis using nucleotide distances. (c) The average number of strains in the reassortant clades with aminoacid distance ≤ τA from the focal node, 〈Nr〉(τA) (red line) is compared to the corresponding average number of strains in the parent clades, 〈N0〉(τA). For τA ≲ 4, both functions increase with τA in an approximately exponential way; we estimate growth rates fr(A) ≈ 0.2 and f0(A) ≈ 0.7, respectively (dashed lines; cf. Eq 1). The growth rate difference s¯A≡f0(A)-fr(A)≈0.5 inferred from distances in aminoacid units is similar to s¯≈0.4 for nucleotide distances; cf. Fig 5c. (PDF) [file ppat.1006685.s003.pdf]

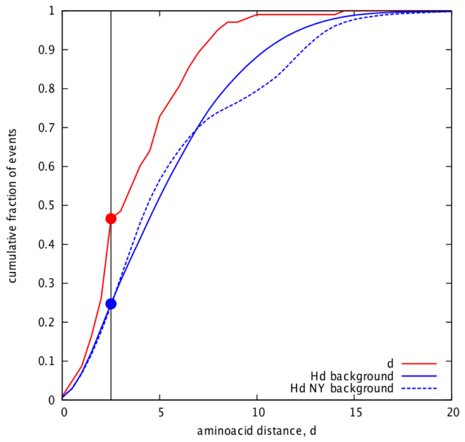

(a)

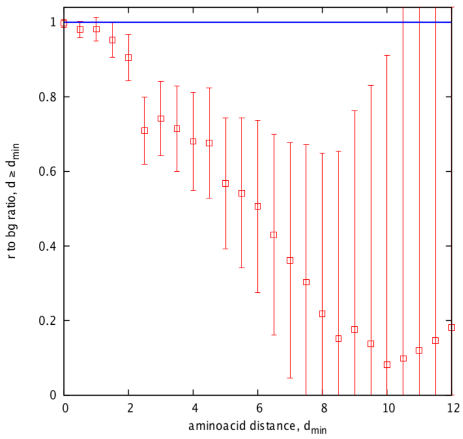

(b)

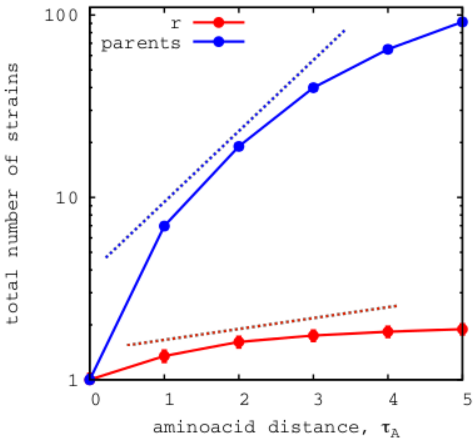

(c)

S3 Fig. Selection inference based on aminoacid distances. (a) The cumulative distribution of mean amino acid distances d between reassortant and parent strains for the HA-NA reassortments in influenza A/H3N2 (red line) is compared to the corresponding distribution of distances for co-circulating strains in the same influenza season (solid blue line) and from the New York area only (dashed blue line). (b) The ratio of reassortment counts to background counts in the interval $d \geq d_{\mathrm{min}}$ (red circles) decreases with increasing lower threshold d_{min} and drops significantly below 1 (blue line). The suppression of reassortment at larger values of d signals distance-dependent negative selection. Bars show statistical errors due to the finite number of inferred reassortments. See Fig. 5 for the same analysis using nucleotide distances. (c) The average number of strains in the reassortant clades with aminoacid distance $\leq \tau_A$ from the focal node, $\langle N_r \rangle(\tau_A)$ (red line) is compared to the corresponding average number of strains in the parent clades, $\langle N_0 \rangle(\tau_A)$. For $\tau_A \lesssim 4$, both functions increase with τ_A in an approximately exponential way; we estimate growth rates $f_r(A) \approx 0.2$ and $f_0(A) \approx 0.7$, respectively (dashed lines; cf. equation 1). The growth rate difference $\bar{s}_A \equiv f_0(A) - f_r(A) \approx 0.5$ inferred from distances in aminoacid units is similar to $\bar{s} \approx 0.4$ for nucleotide distances; cf. Fig. 5c.
