# Supplementary material for: Fitness cost of reassortment in human influenza
Source: PLoS Pathog. 2017 Nov 7;13(11):e1006685. doi: 10.1371/journal.ppat.1006685 (PMC5675378; doi:10.1371/journal.ppat.1006685)
Supplement: S4 Fig — (a) The background distribution P0aa(dHA,dNA) (contour plot) is compared to reassortment counts (red dots). (b) Conditional background distributions P0aa(dHA-dNA|dHA+dNA) (whisker plots) are compared to reassortment counts (red dots). Whisker plots show the 0.25 quantile to the 0.75 quantile of the distribution (blue boxes); the white horizontal line represents the median, vertical bars span the dataset excluding outliers. The width of the bins is chosen to ensure a statistically relevant number of events for each bin. The reassortment data appear more spread in the coordinate dHA − dNA compared to the background (red points are mainly placed outside or at the border of the blue boxes). (PDF) [file ppat.1006685.s004.pdf]

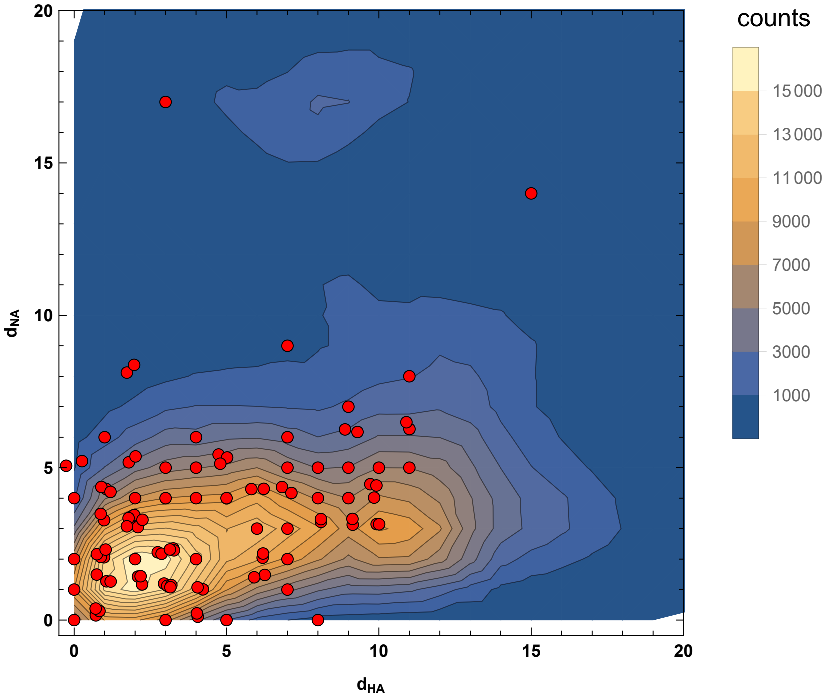

(a)

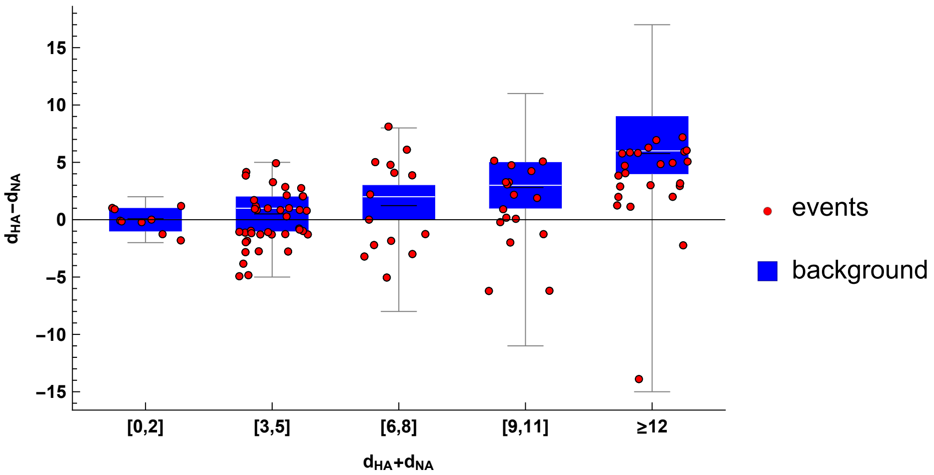

(b)

S4 Fig. Background distribution and reassortment events as a function of the amino acid distances d_{HA} and d_{NA} between strains. (a) The background distribution $P_0^{aa}(d_{\mathrm{HA}}, d_{\mathrm{NA}})$ (contour plot) is compared to reassortment counts (red dots). (b) Conditional background distributions $P_0^{aa}(d_{\mathrm{HA}} - d_{\mathrm{NA}} | d_{\mathrm{HA}} + d_{\mathrm{NA}})$ (whisker plots) are compared to reassortment counts (red dots). Whisker plots show the 0.25 quantile to the 0.75 quantile of the distribution (blue boxes); the white horizontal line represents the median, vertical bars span the dataset excluding outliers. The width of the bins is chosen to ensure a statistically relevant number of events for each bin. The reassortment data appear more spread in the coordinate $d_{\mathrm{HA}} - d_{\mathrm{NA}}$ compared to the background (red points are mainly placed outside or at the border of the blue boxes).
