# Supplementary material for: Fitness cost of reassortment in human influenza
Source: PLoS Pathog. 2017 Nov 7;13(11):e1006685. doi: 10.1371/journal.ppat.1006685 (PMC5675378; doi:10.1371/journal.ppat.1006685)
Supplement: S1 Table — (PDF) [file ppat.1006685.s005.pdf]

δ	**false positives**
1	240
2	53
3	10
4	2
5	0.3
6	0.15

S1 Table. Number of expected false positive reassortment counts as a function of δ (cf. Fig. 3).
