# Supplementary material for: Fitness cost of reassortment in human influenza
Source: PLoS Pathog. 2017 Nov 7;13(11):e1006685. doi: 10.1371/journal.ppat.1006685 (PMC5675378; doi:10.1371/journal.ppat.1006685)
Supplement: S2 Table — Column 2: mean nucleotide distance d between reassortant strain and parent strains. Columns 3–5: representative observed strains in the clades of p, p′ and r, respectively. Each isolate is identified by its number in the online EpiFlu DATABASE (http://www.gisaid.org) identifier (e.g. EPI_ISL_7064 is reported here as 7064). Stars indicate events which are reported in literature with large agreement (S1 Text). (PDF) [file ppat.1006685.s006.pdf]

event	distance, d	parent clade, p	parent clade, p'	reassortant clade, r
1*	11.5	29489,14381,30237,14372,29491	14338,3758,14333	14358
2	8.5	154756	134337,145114,148480,144791,132668	163337
3*	15	6631,8200,166347	8978,8977,9217,5090,8981	8706,6710,6678
4	17	142809,140296,137259,160602,131807	152656,90466,98995,152503,79676	145506
5	12.5	198035	195744,195907,197988,194978,197748	198065
6	12	79650,94769,85831,86055,83861	65125,77838,93878,34977,32310	86052
7	4.5	152192,152613,152818,152498,152358	152291,152817,152255,152392,152775	152686
8	4.5	16068	97156,14327,14323,3750,14330	16000
9*	12	86052	85607,90818,94612,94642,93661	90054
10	7	194156,173986,178004,175204,179355	172767,170296,169683	170296
11	10.5	189816,170207,191802,170197,170701	145483,147970,174249,160586,132668	191800
12	5.5	137758	142809,140296,137259,160602,131807	161491
13	15.5	170266,169497	160279,140419,138011,160321,131805	175187
14	9.5	162098,161905,162096,162135,155890	145483,147970,174249,160586,132668	158821
15*	21	128700,128654	103313	192795
16	11.5	81387,81399,66705	66704,102802,102662,68571,93892	66706
17	11.5	155921	158766,159638,153710,153167,156896	163130
18	7	107846,128072,104125	99876,88040,119705	128044
19	4	194530	193338,193325,195842,195886,197932	197913
20	9	159630,162123,164260,154747	134337,145114,148480,144791,132668	164218
21	13	154746	176988,154023,174836,145111,173207	161320
22	9.5	194642	164815,165579,162145,164711,167825	191815
23	4	195846	197859,194530,197925,197917,197916	197955,197909,197942,197904,197932
24	8.5	152165,152812,152248,152363,152278	143018,83810,87896,89798,81393	152392
25*	6	6679	9235,5017,6471,9177,8630	5015,5032
26	10	15986	98707,117862,30238,14396,33845	90492
27	11	89668,91142,136596,78771,93667	143018,83810,87896,89798,81393	83166
28*	13	115460,115461	111170	115515
29	9	164985,164983,191803,168905,166842	163091,165115,152908,159630,166303	191777
30	4	197888,197905,197964	193338,191710,172747,195246,175213	197930
31	9.5	5109,6508,5696,7057,6355	14254,14247,14253,14252,14266	14249
32	7.5	119713,111411,136406,122660,106394	101916,122601,121525	152345
33	4.5	70811	62803,32221,70811,27632,23843	79673
34	9.5	20566	120392,14384,5728,90490,118003	20572
35	14	152464	152758,152192,96104,152291,90810	152439
36	9	77767,26246,23932,76127	20786,29631,23281,23270	107452
37	6	84613	29908,32258,28882,15889,19060	84614
38	6.5	160943	15483,22763,15558,19707,20684	19896
39	4	191673,194131,172496,169910,176539	193359,172580,173245,169126,170030	170667
40	12	65123	70811,62820,32252,150395,32307	79650,94769,85831,86055,83861
41	6.5	86093	86091,88045,90589	88045
42	25.5	121959,121940,122678,117434,104122	117447,99871,117446,103748,104144	119881
43	8.5	140701	88021,96020,90603,93713,90646	162151
44	5	104109	128049,94718,128048,136373,90646	104134
45	17	191710,170685,170646,191041,176677	134337,145114,157218,148480,156897	191786
46	7	175247	169932,178986,172739,173249,191695	197973
47	19.5	188727	132678,160596,153076,153306,139916	193314
48	5.5	198011,198006	178978,193175,176501,172555,170431	197908
49	17.5	191775	132678,160596,153076,153306,139916	194156
50	10.5	168140,173216,174183,172587,171390	161899,159526,173051	195875,195879
51	3	132678,160596,153076,153306,139916	161899,159526,173051	166226
52	25.5	121931,128651,128649,107858,107855	134337,145114,157218,148480,156897	159618
53	13	90835,86091,119713,109757,133061	118540,93786,85720,88963	85722
54	7	160374,160359,160405,160299	134774,148031,160490,160466,148033	148059
55	9.5	140897	143018,83810,87896,89798,81393	140901
56	11.5	131268,131358,131356,131269,133998	88021,90603,90599,94718,90646	138002
57	6.5	156905	166290,171388,158968,154764,173207	171375
58	8.5	79331	192757	172586

59	11.5	120294,99877	117447,99871,117446,103748,104144	128072
60	6	66079	62803,19055,118061,19712,23843	97483
61	9.5	143015,143014	62803,19055,118061,19712,23843	76700
62	9.5	188727	170724,188755,179340,179369,169915	192181
63	7.5	117460,117461,103744	103248,98651,117449,94724,88040	134331
64	6.5	129899,121972,107830,128027,117655	98651,121258,107852,122974,88040	104113
65	6	171931	170724,188755,179340,179369,169915	168903
66	10	123055,122961,118652,100445,100516	121258,121936,127833,122932,128053	119708
67	8.5	154022,163098,168369,165589,164250	121258,121936,127833,122932,128053	152955
68	5	168931,170309	170724,188755,179340,179369,169915	178093
69	4.5	170638,191812	193338,191710,191674,170724,169915	191813
70	8.5	13724	14410,32482	20286
71	21.5	115303,115354,114867,8696	114610	5665
72	7	173048	147970,174249,134012,192798,148094	173051
73	11	197937,197934	147970,174249,134012,192798,148094	197889
74*	42.5	5022,9235,9198,5466,6674	20810,14305,14327,14323,14321	5009,107554
75	4.5	14137	14137	14187
76	19	16064	14384,90490,154552,90544,14394	70807
77	8	93799,65120,93914,77779,93900	62803,19055,118061,19712,23843	93801
78	6	153942	147970,174249,134012,192798,148094	154770
79	10.5	83173,152458,93666,152193,152600	62803,32221,70811,19055,32251	108212
80	16	191786	168783,170701,167130	179483
81	4.5	6848,20832,25012,25013,22823	113017	8984
82	16	172559	167130,191714,169186	170711
83	5.5	191813	191674,170724,188755,179340,169915	186617
84	16	177539	168783,170701,167130	178965
85	4.5	160950	19055,19712,27632,29908,29574	60739
86	7.5	155910	147970,174249,134012,192798,148094	162113
87	17	175052,179472,174982,190933,193176	176425,173233,171740	191932
88	5	161275	147970,174249,134012,192798,148094	177541
89	14.5	191789	168897,188817,175028,167920,172766	179486
90	12	192166	147970,174249,134012,192798,148094	191968
91	12.5	86052	77951,32286,160994,87188,77762	79326
92	8	8672,8198,114249,114246	5105	114398,114396,110907
93	5	98651,99773,122942	122956,99874,99873,122999,122902	123005
94	13.5	170843	147970,174249,134012,192798,148094	191799
95	8	159133,164603,159141	147970,174249,134012,192798,148094	164216
96	12.5	161480	122956,99874,99873,122999,122902	123069
97	15.5	172583	167961,166459,189816,169089,168899,	175159
98*	14.5	7064,114014	8698,9281,8698,5106	114196
99*	26	16012,90502	9078,5007,5637,5508,9064	107634,107487,107930,107388,6659
100	7	114868	114610	111020
101	13	169946	192798,134337,131805,132579,189622	170734,170324,168100,172756,170703
102	4	85722	152656,90466,98995,152503,79676	85719
103	10.5	142676	152656,90466,98995,152503,79676	142677

S2 Table. List of inferred reassortment events from 1968 to 2015 between HA and NA segments in human influenza A/H3N2. Column 2: mean nucleotide distance d between reassortant strain and parent strains. Columns 3-5: representative observed strains in the clades of p, p' and r, respectively. Each isolate is identified by its number in the online EpiFlu^{TM} DATABASE (http://www.gisaid.org) identifier (e.g. EPI_ISL_7064 is reported here as 7064). Stars indicate events which are reported in literature with large agreement (S1 Text).
