# Supplementary material for: Fitness cost of reassortment in human influenza
Source: PLoS Pathog. 2017 Nov 7;13(11):e1006685. doi: 10.1371/journal.ppat.1006685 (PMC5675378; doi:10.1371/journal.ppat.1006685)
Supplement: S1 Text — (PDF) [file ppat.1006685.s007.pdf]

S1 Text. Comparison with reassortment reported in literature. As highlighted by Pinsent et al [36], there are few intra-subtype reassortment events which are reported in literature with large consensus. Among these, a major event occurred between isolates co-circulating in New York in the early 2000s is well-documented. This event, which involved also strains with mixed HA and NA segments, has been related to the jump from A/Sydney/5/1997-like to A/Fujian/411/2002-like antigenic clusters (Holmes et al. [13]). Event number 3 in S2 Table coherently represents the event occurred between the main group of viruses sampled in the 1999–2000 season (A/New York/315/1999-like viruses) and the isolate A/New York/177/1999 (which appears genetically close to other two strains collected in a different geographical region: A/Memphis/59/1999 and A/Netherlands/051/2000). This event gave origin to the strains A/New York/137/1999 and A/New York/138/1999 (green colored in [13]), that diverge from the clade leading in 1999-2000 season. Event number 74 refers to the appearance of the Fujian/02-like reassortants A/New York/198/2003 and A/New York/199/2003 (referred in [13] as Clade B and colored in yellow). We report this event with a large d between the parents, which is consistent with the clear separation in NA phylogeny with respect to the main circulating clade (Clade A, light blue in [13]). The remaining events marked with a star in S2 Table refer to a consensus list of reassortments provided in [36], including two large events occurred in the Netherlands (events 98 and 99, Westgeest et al [37]) and in Hong Kong. In addition, we have found that some of the events reported in the referenced studies as independent (Rabadan et al [28], [36], [37]) are grouped in our analysis as part of the same reassortment event. In addition, we apply restrictive criteria to select the sequences that are included in our analysis, in order to both avoid over-counting and ensure that the reported events are as clean as possible. This is essential to prevent non controllable factors confounding the results on the main biological object of this study, i.e. inference of selection. As a result, some of the minor events reported in other studies include sequences which are not in our database, and therefore cannot be reported here. However, the overall agreement with the other studies, signaled by the presence of the majority of the reported events in our list, guarantees that we are able to detect at least a large fraction of the real events.
